# Postoperative decreased levels of D-dimer in patients with gynecologic cancer with enoxaparin and fondaparinux thromboprophylaxis

**DOI:** 10.3892/mco.2013.120

**Published:** 2013-05-14

**Authors:** JUNICHI KODAMA, NORIKO SEKI, CHIKAKO FUKUSHIMA, TOMOYUKI KUSUMOTO, KEIICHIRO NAKAMURA, YUJI HIRAMATSU

**Affiliations:** 1 Department of Obstetrics and Gynecology, Hiroshima City Hospital, Naka-ku, Hiroshima 730-8518;; 2 Department of Obstetrics and Gynecology, Okayama University Graduate School of Medicine, Kita-ku, Okayama 700-8558, JapanDentistry and Pharmaceutical Sciences, Kita-ku, Okayama 700-8558, Japan

**Keywords:** gynecologic cancer, venous thromboembolism, D-dimer, enoxaparin, fondaparinux

## Abstract

The purpose of the present study was to evaluate the effects of enoxaparin (ENO) and fondaparinux (FPX) on postoperative plasma D-dimer levels and risk factors associated with postoperative venous thromboembolism (VTE) and pulmonary thromboembolism (PTE) in patients with gynecologic cancer. For this study, 434 patients with gynecologic cancer were recruited and a surgical treatment strategy was employed. Plasma D-dimer levels were measured prior to surgery, as well as on a schedule up to 3 weeks postoperatively and again after day 28. Patients with clinical signs and elevation of the plasma D-dimer level underwent multidetector row computed tomography. The D-dimer value was significantly lower in patients with ENO or FPX on postoperative days 3–10 compared to patients with gynecologic cancers who were not receiving ENO or FPX. The D-dimer value was significantly lower in patients with FPX compared to patients with ENO on postoperative days 5–7. The D-dimer value on postoperative day 3, the use of erythropoiesis-stimulating agents (ESAs), advancing age and non-O blood group were independent risk factors for postoperative VTE. The D-dimer value on postoperative day 3 and the use of ESAs were independent risk factors for postoperative PTE. The postoperative D-dimer value was significantly lower in patients with gynecologic cancer who were administered ENO or FPX compared to patients were not administered either ENO or FPX. The use of ESAs and high plasma D-dimer levels on postoperative day 3 were independent risk factors for postoperative VTE and PTE.

## Introduction

Cancer is a widely accepted risk factor for venous thromboembolism (VTE) and, to a lesser extent, arterial thrombosis. This risk is attributed to factors such as the expression of prothrombotic factors by tumors, tumor compression of vessels, inflammatory host response to malignancies, immobility, surgery, indwelling central venous catheters and certain antitumor therapies (1). In cancer patients undergoing surgery, VTE is the most common cause of mortality in the first 30 postoperative days (2). VTE is also a common complication of surgery for gynecologic cancer and gynecologic malignancies are classified as the highest risk group (3,4). It has been reported that 90% of pulmonary thromboembolism (PTE) resulted from deep venous thromboses (DVT). Kearon (5) reported that, in a large number of cases, VTE occurred between the intraoperative period and the first 3 postoperative days and that thromboemboli in many of these patients resolved spontaneously (50% resolved within 72 h). The risk of progression of postoperative VTE appears to be greater if the initial thrombosis is large and if there are continuing risk factors for thrombosis. The risk of symptomatic VTE is generally the highest within the first 2 weeks after surgery.

D-dimer, a marker of the hypercoagulable state, is a stable end product of fibrin degradation. D-dimer levels increase because of fibrin formation and fibrinolysis. Plasma D-dimer measurement has been widely used in the screening for VTE. The time course of changes in plasma D-dimer levels following surgery has not been defined, although a recent study clarified the time course associated with changes in plasma D-dimer levels after surgery in patients with gynecologic cancer (6). The European Society for Medical Oncology (ESMO) Clinical Practice Guidelines recommends the use of low-molecular-weight heparin (LMWH) [e.g., enoxaparin (ENO) 4,000 units], unfractionated heparin (UFH) 5,000 units, 3 times daily, or fondaparinux (FPX) 2.5 mg in patients undergoing major cancer surgery (7). ENO and FPX were approved by the Japanese Ministry of Health, Labor and Welfare for use in the prevention of VTE in 2008 and 2009, respectively, and have since been routinely used postoperatively for VTE prophylaxis. No studies have evaluated the efficacy of ENO or FPX in terms of postoperative D-dimer levels in patients with gynecologic cancer. The purpose of the present study was to evaluate the effects of ENO and FPX on postoperative plasma D-dimer levels in patients with gynecologic cancer. Furthermore, pre-, intra- and postoperative parameters, including use of ENO or FPX usage, were investigated as possible risk factors for postoperative VTE or PTE.

## Materials and methods

### Study population

For this study, 434 patients with invasive gynecologic cancer, who underwent surgery at Okayama University Hospital (Okayama, Japan) between August, 2005 and August, 2011 were recruited. Written informed consent was obtained from all patients. The median age of the patients was 55 years (range, 16–85 years). Patients with a range of gynecologic cancers were enrolled, including 118 ovarian, 204 endometrial, 146 cervical and 3 vulvar cancers. Patients with preoperative VTE were excluded from the study. This study was approved by the ethics committee of Okayama University Hospital.

### Measurement of D-dimer levels and detection of VTE

The plasma D-dimer level was measured prior to surgery and on postoperative days 0, 1, 3, 5, 7, 10, 14 and 21, and a final level was measured after day 28. The D-dimer level was measured with a latex photometric immunoassay system using LPIA-Ace D-D dimer II (Mitsubishi Chemical Medience Corporation, Tokyo, Japan) as the reagent. Inter-assay variability (coefficient of variation) was <10%. Preoperative plasma D-dimer levels were used as baseline and the postoperative cut-off values were set at 10–15 *μ*g/ml, in accordance with previous studies (6). Following surgery, primary screening for VTE was performed using meticulous evaluation of the clinical signs and elevation of the plasma D-dimer level. The clinical signs of VTE evaluated in this study were leg swelling, tenderness along the distribution of deep veins, acute cardiovascular dysfunction, dyspnea, chest pain and loss of consciousness. Patients whose plasma D-dimer concentration exceeded the pre-set cut-off values (10–15 *μ*g/ml) and patients who showed clinical signs of VTE underwent CT scanning of the chest, abdomen and lower extremities by using MDCT (Toshiba).

### Demographic data

Preoperative autologous blood collection was performed to minimize homologous blood transfusion in selected cases of radical hysterectomy and/or para-aortic lymphadenectomy. Erythropoiesis-stimulating agents (ESAs) were preferentially used to correct anemia associated with the collection of autologous blood. Preoperative autologous blood was obtained from 227 patients and ESAs were administered to 180 of these patients. The patients received general anesthesia. Calf-length external sequential compression devices (SCD) were placed on both legs at the start of surgery in all the patients; the use of these devices continued postoperatively. In all, 270 patients underwent chemical anticoagulation: 107 patients received UFH (10,000 U/day) following surgery, 51 patients received FPX (2.5 mg/day) and 112 patients received ENO (4,000 units) after the surgery. UFH, ENO and FPX were continued for a median of 5 days (range, 3–23 days), 10 days (range, 1–15 days) and 10 days (range, 3–20 days), respectively. UFH was used at the surgeon’s discretion prior to the regulatory approval of the other agents (e.g., prior to 2008) and ENO and FPX were routinely used subsequent to 2009. Age, history, body-mass index, hematologic data, the results of biochemical tests, type of surgery, intraoperative findings and postoperative information were obtained from the medical records of the patients.

### Statistical analysis

The Mann-Whitney U test was used to compare plasma D-dimer levels or postoperative day of VTE detection in the groups. The Chi-square test, Mann-Whitney U test and logistic regression analysis were used to investigate the relationship between various variables and the occurrence of VTE or PTE. P<0.05 was considered to indicate a statistically significant difference.

## Results

### Postoperative VTE and PTE

When the groups of patients were combined, VTE was detected in 31 (7.1%) patients on postoperative days 1–21 (median, day 7). The median postoperative day of VTE detection was significantly later in the case of patients treated with ENO or FPX (median, day 11) compared to patients who did not receive ENO or FPX (median, day 5.5) (P=0.028). The incidence of VTE in patients with ovarian, endometrial and cervical cancer was 7.4, 7.7 and 5.6%, respectively (Table I). PTE was found in 14 patients (3.2%). The incidence of PTE in patients with ovarian, endometrial and cervical cancer was 3.2, 3.1 and 3.5%, respectively (Table I). PTE was clinically symptomatic (dyspnea or chest pain) in 3 patients, but not fatal in any patient. A substantial number of thrombi and PTEs were treated using anticoagulation therapy with parenteral UFH followed by oral warfarin. Insertion of an inferior vena cava filter was required in 5 cases.

### Time course of changes in postoperative plasma D-dimer levels

**Figure 1.**
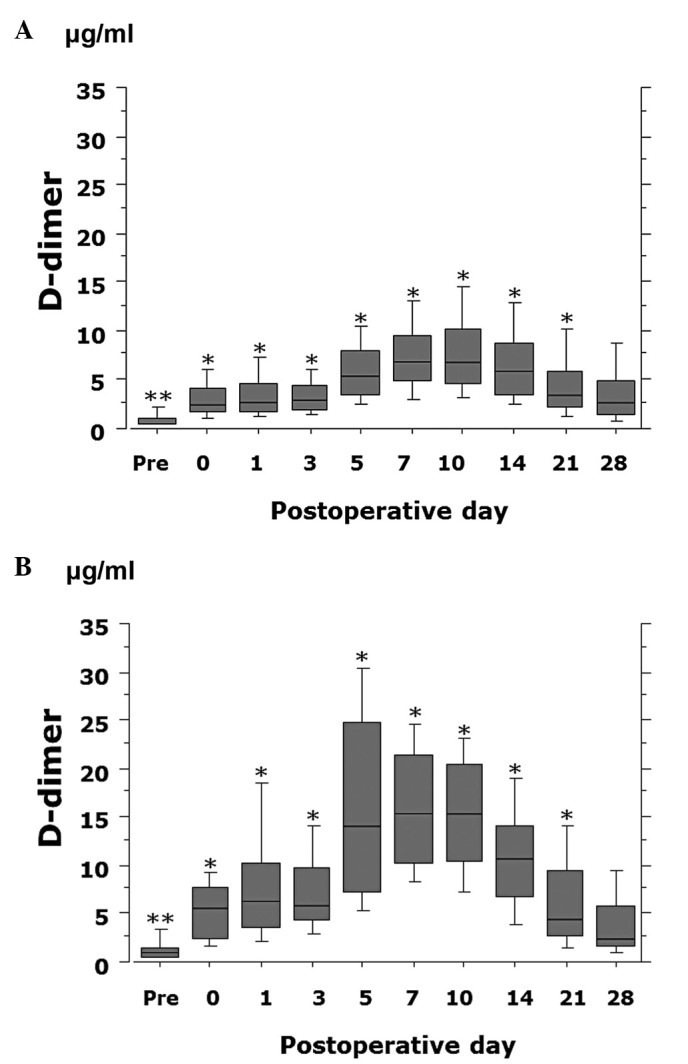
Plasma D-dimer levels with respect to time in venous thromboembolism (VTE)-positive and VTE-negative patients. (A) Time course of changes in plasma D-dimer levels in patients who did not have VTE (n=403). (B) Time course of changes in plasma D-dimer levels in patients who had VTE (n=31). Median values are indicated by a horizontal line inside the box. The lower edge of the whisker, lower end of the box, upper end of the box and upper edge of the whisker represent the 10th, 25th, 75th and 90th percentiles, respectively. *P<0.0001, **P=0.047.

**Figure 2.**
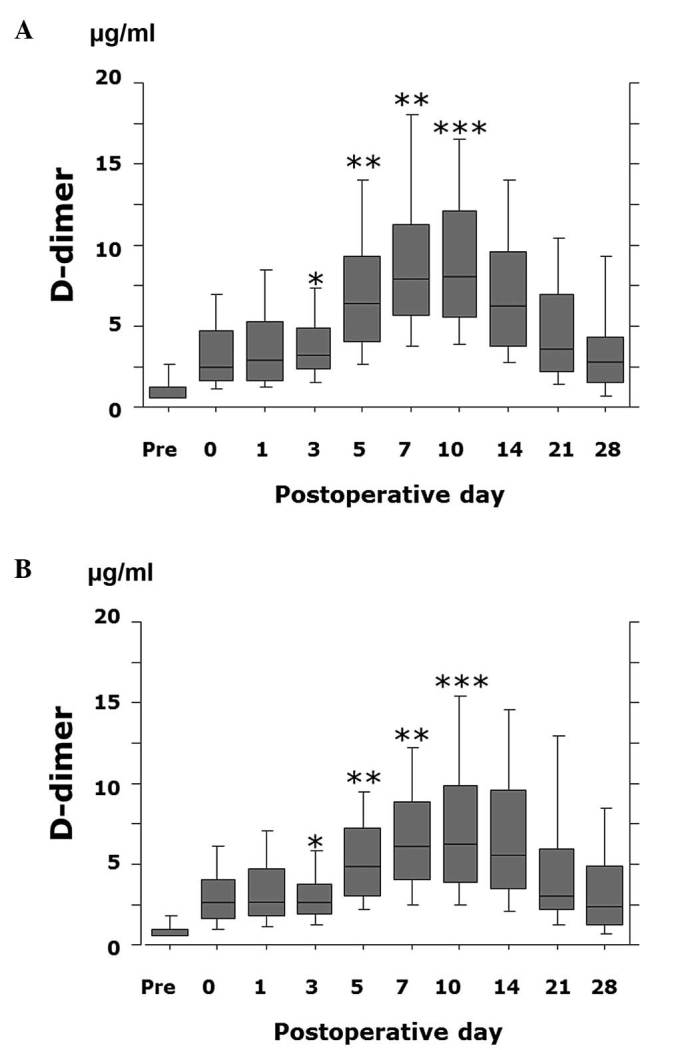
Plasma D-dimer levels with respect to time in patients with or without chemoprophylaxis. (A) The time course of changes in plasma D-dimer levels in patients not receiving enoxaparin (ENO) or fondaparinux (FPX) prophylaxis (n=271). (B) The time course of changes in plasma D-dimer levels in patients receiving ENO or FPX prophylaxis (n=163). *P=0.0004, **P<0.0001, ***P=0.0009.

**Figure 3.**
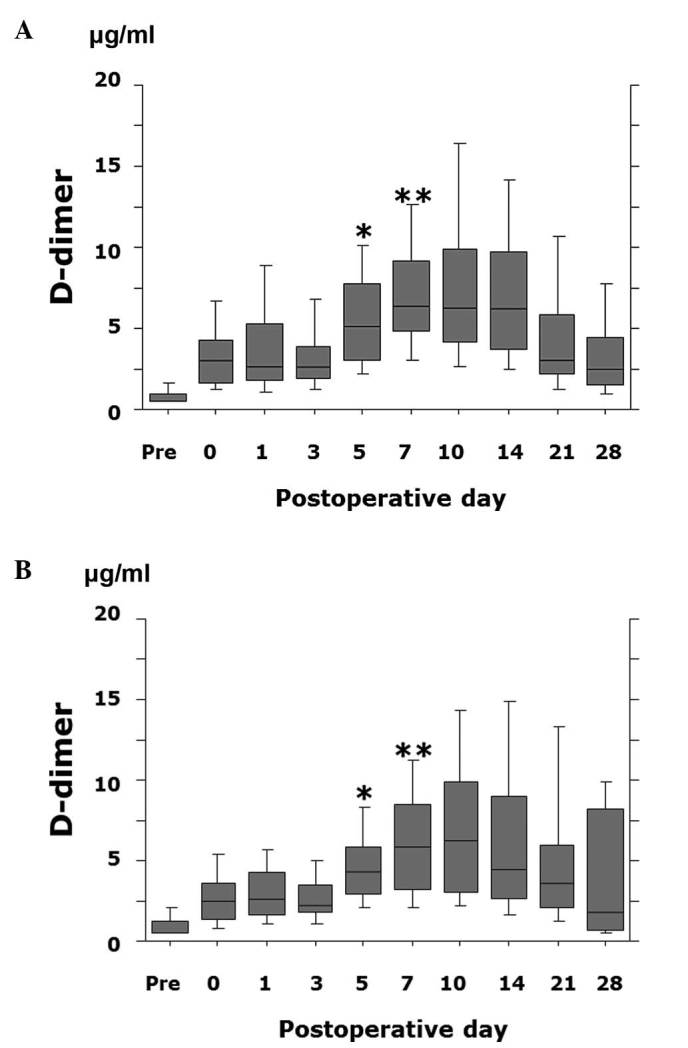
Plasma D-dimer levels with respect to time in patients receiving enoxaparin (ENO) or fondaparinux (FPX) chemoprophylaxis. (A) The time course of changes in plasma D-dimer levels in patients receiving ENO prophylaxis (n=112). (B) The time course of changes in plasma D-dimer levels in patients receiving FPX prophylaxis (n=51). *P=0.028, **P=0.049.

The plasma D-dimer value gradually increased postoperatively, peaked on postoperative days 7–10 and then decreased. The D-dimer value significantly differed between VTE-positive and -negative patients preoperatively (P=0.047) and on postoperative day 0–21 (P<0.0001) (Fig. 1). The D-dimer value was significantly lower on postoperative day 3 (P=0.0004), days 5–7 (P<0.0001) and day 10 (P=0.0009) in patients receiving ENO or FPX compared to patients not treated with ENO or FPX (Fig. 2). In addition, the D-dimer value was significantly lower on postoperative day 5 (P=0.028) and day 7 (P=0.049) in patients receiving FPX compared to patients treated with ENO (Fig. 3).

### Risk factors for postoperative VTE and PTE

Results of univariate analysis indicated that the D-dimer value on postoperative day 3, non-O blood group, the use of ESAs, length of surgery, the extent of lymph node dissection, age and the preoperative D-dimer value were significant risk factors for postoperative VTE (Tables II and III). The use of ENO or FPX was not associated with the incidence of VTE. Logistic regression multivariate analysis revealed that the D-dimer value on postoperative day 3, the use of ESAs, advanced age and non-O blood group were independent risk factors for predicting the occurrence of postoperative VTE (Table IV). The D-dimer value on postoperative day 3, the use of ESAs and not receiving treatment with ENO or FPX were significant risk factors for postoperative PTE (Tables V and VI). Logistic regression multivariate analysis revealed that the D-dimer value on postoperative day 3 and the use of ESAs were independent risk factors for predicting the occurrence of postoperative PTE (Table VII).

## Discussion

The present study measured D-dimer values longitudinally in patients in whom sub-clinical VTE prior to surgery was ruled out. These results showed that plasma D-dimer values were significantly higher in VTE-positive patients compared to VTE-negative patients preoperatively and on postoperative days 0–21, but not on day 28.

The changes in postoperative D-dimer levels demonstrated the same pattern in patients treated with or without UFH, although 5,000 units of UFH twice daily was commonly used for a median of 5 days in cases requiring a more aggressive surgical procedure (data not shown). Findings of a previous study have shown that treatment with 5,000 units of UFH, 3 times daily, significantly decreased the incidence of DVT in gynecologic cancer patients, whereas this was not the case with treatment twice daily (3). Recently, ENO and FPX were approved for use as prophylaxis in Japanese patients undergoing abdominal or pelvic surgery. Following approval, prophylaxis with ENO or FPX for a median of 10 days has been routinely used at this institution, (as well as other institutions). To the best of our knowledge this is the first study to demonstrate a significantly lower D-dimer value on postoperative days 3–10 in patients receiving chemoprophylaxis with ENO or FPX compared to patients receiving prophylaxis with SCDs or SCDs + UFH. These results suggest that the decrease in the D-dimer level is a result of fibrin formation and fibrinolysis due to administration of ENO or FPX. Furthermore, this study demonstrated that the D-dimer value was significantly lower in patients receiving FPX compared to those treated with ENO on postoperative days 5 and 7. Therefore, 2.5 mg of FPX may have stronger anticoagulant properties than 4,000 units of ENO. Specifically, a subgroup analysis in the PEGAS study shed some light on the efficacy of FPX relative to LMWH for post-surgical thromboprophylaxis in patients with cancer (8).

The result of our previous study (9) demonstrated that the incidence of preoperative VTE was significantly higher in patients with ovarian cancer than in those with other types of gynecologic cancer. Unlike preoperative VTE, ovarian cancer was not significantly associated with postoperative VTE. In the present study population, VTE was found in 7.9% of the patients after the surgery. Owing to the high risk of postoperative VTE, this study also attempted to identify a number of risk factors that may be useful in identifying gynecologic cancer patients who are at even higher risk of developing postoperative VTE. In contrast to results of the previous study, results of the present study showed that a number of factors were associated with VTE in the overall study population. Age was an independent risk factor in the current study population, as well as a high plasma D-dimer level on postoperative day 3, the use of ESAs and non-O blood group, which have been previously reported (6). These results demonstrate that the use of ENO or FPX significantly decreased the incidence of PTE, although therapy with these agents did not reduce the incidence of VTE. The lack of statistical significance detected for VTE is likely due to insufficient power to show a difference in VTE rates. This study also showed that a high plasma D-dimer level on postoperative day 3 and the use of ESAs were independent risk factors for PTE.

The use of ESAs was demonstrated to be an important risk factor for postoperative VTE and PTE. ESAs are biosynthetic forms of erythropoietin, with similar biochemical structure and biologic effects to those of erythropoietin (10). In this study, preoperative autologous blood collection was undertaken to minimize homologous blood transfusion in cases of radical hysterectomy and/or para-aortic lymphadenectomy and ESAs were preferentially used to correct anemia associated with the collection of autologous blood. Analysis of the safety profile of ESAs for patients prior to their going spinal surgery or open radical retropubic prostatectomy revealed that the use of this hormone does not increase the risk of thromboembolic events (11,12). An open-level, randomized, parallel-group study was conducted to confirm the safety and efficacy of epoetin α (PROCRIT) administered perioperatively vs. the standard of care in blood conservation in subjects undergoing major elective spinal surgery, available online at http://clinicaltrial.gov/show/NCT00211146. This observation led to the FDA issuing black box warnings for ESAs, stating that ‘Perisurgery: Due to increased risk of DVT, DVT prophylaxis is recommended’. The ESMO clinical practice guideline states that the use of ESAs should be carefully reconsidered in the case of patients at a high risk of thromboembolic events, such as those undergoing surgery (13). These results clearly show that the use of ESAs is an independent risk factor for the development of VTE and PTE after surgery in patients with gynecologic cancer. Additional research on the safety of ESAs is required to confirm these results.

Owing to the suppression of the D-dimer value until postoperative day 10 in patients undergoing treatment with ENO or FPX, the median postoperative day of VTE detection was day 11 in the present study. This observation suggests that thromboprophylaxis reduces the incidence of VTE and delays the occurrence of VTE. In the Enoxacan II study, a double-blinded, multicenter, clinical trial of patients undergoing open abdominal or pelvic cancer surgery, the incidence of VTE significantly decreased from 12 to 4.8% when patients received inpatient thromboprophylaxis for 27–31 days (14). The ESMO clinical practice guidelines recommend that cancer patients undergoing elective major abdominal or pelvic surgery should receive in-hospital and post-discharge prophylaxis with LMWH for up to 1 month after surgery (7). The present study suggests that extended-duration prophylaxis may be considered for patients with gynecologic cancer and other risk factors such as advanced age, a high plasma D-dimer level on postoperative day 3, the use of ESAs and non-O blood group. However, a systematic review concluded that extended prophylaxis reduced asymptomatic VTE without decreasing the risk of death at 3 months and that the evidence for extended-duration regimens was limited and of poor quality (15). More studies are required to determine an optimal, cost-effective chemoprophylaxis regimen.

The current findings demonstrate that the postoperative D-dimer value was significantly lower in patients with gynecologic cancer receiving ENO or FPX prophylaxis. Furthermore, through multivariate analysis, the use of ESAs and the presence of a high plasma D-dimer level on postoperative day 3 were shown to be independent risk factors for postoperative VTE and PTE.

## Figures and Tables

**Table I.** Incidence of postoperative VTE and PTE in patients with gynecologic cancer.

Cancer type	VTE, n (%)	PTE, n (%)
Ovarian (n=95)	7 (7.4)	3 (3.2)
Endometrial (n=194)	15 (7.7)	6 (3.1)
Cervical (n=142)	8 (5.6)	5 (3.5)
Vulvar (n=3)	1 (33.3)	0 (0.0)

VTE, venous thromboembolism; PTE, pulmonary thromboembolism.

**Table II.** Univariate analysis of preoperative predictors of VTE.

Variables	VTE (−) (n=403)	VTE (+) (n=31)	P-value
Age (years)			0.023
Median (range)	55 (23–85)	58 (32–75)	
BMI (kg/m^2^)			0.827
Median (range)	22.9 (13.5–46.5)	22.3 (15.0–32.0)	
Comorbidity	103 (25.6)	12 (38.7)	0.110
Smoking	38 (9.4)	0 (0.0)	0.074
Blood group			0.004
O	105 (26.1)	1 (3.3)	
Non-O	298 (73.9)	30 (96.7)	
Diagnosis group			0.296
Ovarian	88 (21.8)	7 (22.6)	
Endometrial	179 (44.4)	15 (48.4)	
Cervical	134 (33.3)	8 (25.8)	
Vulvar	2 (0.5)	1 (3.2)	
Usage of ESAs	160 (39.7)	20 (64.5)	0.007
Pre-operative D-dimer (*μ*g/ml)			0.030
Median (range)	0.5 (0.5–24.1)	0.9 (0.5–6.2)	
White blood cell (/mm^3^)			0.067
Median (range)	6360 (2090–18300)	5800 (3700–9890)	
Hemoglobin (g/dl)			0.813
Median (range)	12.8 (2.9–15.7)	12.9 (9.0–15.9)	
Platelet (/mm^3^)			0.199
Median (range)	27.8 (3.4–97.1)	26.2 (13.6–43.0)	
CRP (mg/dl)			0.971
Median (range)	0.06 (0.0–14.0)	0.06 (0.0–7.5)	
PT-INR			0.123
Median (range)	0.93 (0.79–1.44)	0.91 (0.84–1.10)	
APTT			0.890
Median (range)	30.5 (21.2–53.2)	30.5 (20.0–43.3)	

Values are median (range) or n (%). P-values were determined using the Mann-Whitney U test or Chi-square test. VTE, venous thromboembolism; BMI, body mass index; ESAs, erythropoiesis-stimulating agents; CRP, C-reactive protein; PT-INR, prothrombin time-international normalized ratio; APTT, activated partial thromboplastin time.

**Table III.** Univariate analysis of intra-/postoperative predictors of VTE.

Variables	VTE (−) (n=403)	VTE (+) (n=31)	P-value
Type of hysterectomy			0.4180
Not removed	37 (9.2)	1 (3.2)	
SH	206 (51.1)	15 (48.4)	
RH	160 (39.7)	15 (48.4)	
Extent of lymphadenectomy			0.0220
None	48 (11.9)	1 (3.2)	
Pelvic	248 (61.5)	15 (48.4)	
Pelvic-paraaortic	107 (26.6)	15 (48.4)	
Operation time (min)			0.2290
Median (range)	300 (140–455)	275 (40–470)	
Blood loss (ml)			0.3490
Median (range)	460 (0–4790)	530 (50–7000)	
Blood transfusion	340 (84.4)	25 (80.6)	0.1300
Usage of anticoagulant			0.1610
None/UFH	248 (61.5)	23 (74.2)	
ENO/FPX	155 (38.5)	8 (25.8)	
D-dimer (*μ*g/ml) (day 3)			
Median (range)	2.8 (0.5–24.2)	5.8 (1.6–21.5)	<0.0001

Values are median (range) or n (%). P-values were determined using the Mann-Whitney U test or Chi-square test. VTE, venous thromboembolism; SH, simple hysterectomy; RH, radical hysterectomy; UFH, unfractionated heparin; ENO, enoxaparin; FPX, fondaparinux.

**Table IV.** Stepwise multivariate logistic regression analysis for predictors of VTE.

Variables	Comparison	Multivariate		
		Odds ratio	P-value	95% CI
Age	Old:Young	1.060	0.010	1.014–1.109
Blood type	Non-O: O	12.801	0.017	1.584–103.463
Use of ESAs	(+):(−)	4.183	0.003	1.610–10.970
Preoperative D-dimer	High: Low	1.067	0.529	0.871–1.307
Extent of lymphadenectomy	Pelvic: None	8.912	0.185	0.571–138.985
	Pelvic + paraaortic: None	10.496	0.099	0.643–171.427
Operation time	Long: Short	1.003	0.333	0.997–1.008
Postoperative D-dimer (day 3)	High: Low	1.300	<0.001	1.162–1.454

VTE, venous thromboembolism; CI, confidence interval; ESAs, eryrhropoiesis-stimulating agents.

**Table V.** Univariate analysis of preoperative predictors of PTE.

Variables	PTE (−) (n=420)	PTE (+) (n=14)	P-value
Age (years)			0.412
Median (range)	55 (16–85)	58 (32–75)	
BMI (kg/m^2^)			0.866
Median (range)	22.9 (13.5–46.5)	22.3 (15.0–32.0)	
Comorbidity	111 (26.4)	4 (28.6)	0.858
Smoking	38 (9.0)	0 (0.0)	0.239
Blood group			0.126
O	105 (25.0)	1 (7.1)	
Non-O	315 (75.0)	13 (92.9)	
Diagnosis group			0.845
Ovarian	92 (21.8)	3 (21.4)	
Endometrial	189 (44.4)	5 (35.7)	
Cervical	136 (33.3)	6 (42.9)	
Vulvar	3 (0.5)	0 (0.0)	
Use of ESAs	169 (40.2)	11 (78.6)	0.004
Pre-operative D-dimer (*μ*g/ml)			0.105
Median (range)	0.5 (0.5–24.1)	0.9 (0.5–6.2)	
White blood cell (/mm^3^)			0.844
Median (range)	6360 (2090–18300)	5800 (3700–9890)	
Hemoglobin (g/dl)			0.427
Median (range)	12.8 (2.9–15.7)	12.9 (9.0–15.9)	
Platelet (/mm^3^)			0.674
Median (range)	27.8 (3.4–97.1)	26.2 (13.6–43.0)	
CRP (mg/dl)			0.994
Median (range)	0.06 (0.0–14.0)	0.06 (0.0–7.5)	
PT-INR			0.328
Median (range)	0.93 (0.79–1.44)	0.91 (0.84–1.10)	
APTT			0.200
Median (range)	30.5 (21.2–55.2)	30.5 (20.0–43.3)	

Values are median (range) or n (%). P-values were determined using the Mann-Whitney U test or Chi-square test. PTE, pulmonary thromboembolism; BMI, body mass index; ESAs, erythropoiesis-stimulating agents; CRP, C-reactive protein; PT-INR, prothrombin time-international normalized ratio; APTT, activated partial thromboplastin time.

**Table VI.** Univariate analysis of intra-/post-operative predictors of PTE.

Variables	PTE (−) (n=420)	PTE (+) (n=14)	P-value
Type of hysterectomy			0.412
Not removed	40 (9.5)	0 (0.0)	
SH	215 (51.2)	7 (50.0)	
RH	165 (39.3)	7 (50.0)	
Extent of lymphadenectomy			0.249
None	48 (11.9)	1 (3.2)	
Pelvic	248 (61.5)	15 (48.4)	
Pelvic-paraaortic	107 (26.6)	15 (48.4)	
Operation time (min)			0.098
Median (range)	300 (140–455)	275 (40–470)	
Blood loss (ml)			0.239
Median (range)	460 (0–4790)	530 (50–7000)	
Blood transfusion	354 (84.3)	11 (78.6)	0.581
Use of anticoagulant			0.018
None/UFH	258 (61.6)	13 (92.9)	
ENO/FPX	162 (38.4)	1 (7.1)	
D-dimer (*μ*g/ml) (Day 3)			
Median (range)	2.8 (0.5–24.2)	5.8 (1.6–21.5)	<0.0001

Values are median (range) or n (%). P-values were determined using the Mann-Whitney U test or Chi-square test. PTE, pulmonary thromboembolism; SH, simple hysterectomy; RH, radical hysterectomy; UFH, unfractionated heparin; ENO, enoxaparin; FPX, fondaparinux.

**Table VII.** Stepwise multivariate logistic regression analysis for predictors of PTE.

Variables	Comparison	Multivariate		
		Odds ratio	P-value	95% CI
Use of ESAs	(+): (−)	4.129	0.0350	1.108–15.389
Use of anticoagulants	ENO/FPX: None/UFH	0.190	0.1170	0.024–1.517
Postoperative D-dimer (day 3)	High: Low	1.222	0.0002	1.102–1.356

PTE, pulmonary thromboembolism; CI, confidence interval; ESAs, eryrhropoiesis-stimulating agents; ENO, enoxaparin; FPX, fondaparinux; UFH, unfractionated heparin.

## References

[1] Lin A, Ryu J, Harvey D, Sieracki B, Scudder S, Wun T (2006). Low-dose warfarin does not decrease the rate of thrombosis in patients with cervix and vulvo-vaginal cancer treated with chemotherapy, radiation, and erythropoietin. Gynecol Oncol.

[2] Bradley CT, Brasel KJ, Miller JJ, Pappas SG (2010). Cost-effectiveness of prolonged thromboprophylaxis after cancer surgery. Ann Surg Oncol.

[3] Einstein MH, Pritts EA, Hartenbach EM (2007). Venous thromboembolism prevention in gynecologic cancer surgery: a sysytematic review. Gynecol Oncol.

[4] http://www.nccn.org/professionals/physician_gls/f_guidelines.asp.

[5] Kearon C (2003). Duration of venous thromboembolism prophylaxis after surgery. Chest.

[6] Kodama J, Seki N, Masahiro S (2010). D-dimer level as a risk factor for postoperative venous thromboembolism in Japanese women with gynecologic cancer. Ann Oncol.

[7] Mandala M, Falanga A, Rolia F (2010). Venous thromboembolism in cancer patients: ESMO Clinical Practice Guidelines for the management. Ann Oncol.

[8] Agnelli G, Bergqvist D, Cohen AT, Gallus AS, Gent M (2005). Randomized clinical trial of postoperative fondaparinux versus perioperative dalteparin for prevention of venous thromboembolism in high-risk abdominal surgery. Br J Surg.

[9] Kodama J, Seki N, Fukushima C (2013). Elevated preoperative plasma D-dimer levels and the incidence of venous thromboembolism in Japanese females with gynecological cancer. Oncol Lett.

[10] Egrie JC, Strickland TW, Lane J (1986). Characterization and biological effects of recombinant human erythropoietin. Immunobiology.

[11] Colomina MJ, Bagó J, Pellisé F, Godet C, Villanueva C (2004). Preoperative erythropoietin in spine surgery. Eur Spine J.

[12] Lepor H, Lipkin M, Slova D (2010). The preoperative use of erythropoietin stimulating proteins prior to radical prostatectomy is not associated with increased cardiovascular or thromboembolic morbidity or mortality. Urology.

[13] Schrijvers D, De Samblanx H, Rolia F (2010). Eruthropoiesis-stimulating agents in the treatment of anaemia in cancer patients: ESMO Clinical Practice Guidelines for use. Ann Oncol.

[14] Bergqvist D, Agnelli G, Cohen AT (2002). Duration of prophylaxis against venous thromboembolism with enoxaparin after surgery for cancer. N Engl J Med.

[15] Akl EA, Terrenato I, Barba M, Sperati F, Muti P, Schünemann HJ (2008). Extended perioperative thromboprophylaxis in patients with cancer. A systematic review. Thromb Haemost.

